# Mucosal-Associated Invariant T Cells: New Insights into Antigen Recognition and Activation

**DOI:** 10.3389/fimmu.2017.01540

**Published:** 2017-11-10

**Authors:** Xingxing Xiao, Jianping Cai

**Affiliations:** ^1^State Key Laboratory of Veterinary Etiological Biology, Key Laboratory of Veterinary Parasitology of Gansu Province, Lanzhou Veterinary Research Institute, Chinese Academy of Agricultural Sciences, Lanzhou, China; ^2^Jiangsu Co-Innovation Center for Prevention and Control of Animal Infectious Diseases and Zoonoses, Yangzhou, China

**Keywords:** mucosal-associated invariant T cells, antigens, recognition, activation, diseases

## Abstract

Mucosal-associated invariant T (MAIT) cells, a novel subpopulation of innate-like T cells that express an invariant T cell receptor (TCR)α chain and a diverse TCRβ chain, can recognize a distinct set of small molecules, vitamin B metabolites, derived from some bacteria, fungi but not viruses, in the context of an evolutionarily conserved major histocompatibility complex-related molecule 1 (MR1). This implies that MAIT cells may play unique and important roles in host immunity. Although viral antigens are not recognized by this limited TCR repertoire, MAIT cells are known to be activated in a TCR-independent mechanism during some viral infections, such as hepatitis C virus and influenza virus. In this article, we will review recent works in MAIT cell antigen recognition, activation and the role MAIT cells may play in the process of bacterial and viral infections and pathogenesis of non-infectious diseases.

## Introduction

T lymphocyte activation is best explained by conventional T cells using a very diverse repertoire of T cell receptors (TCRs), which recognize various antigenic peptides presented in the context of classical major histocompatibility complex (MHC) class I or class II molecules (1, 2). This response is adaptive or acquired and is different from the innate immune response, which is MHC and TCR independent. In humans and mice, however, there are also other important groups of T cells, known as non-conventional T cells, which can be activated in a mechanism distinct from conventional T cells. Unlike conventional T cells, which recognize peptide antigens, invariant natural killer T (iNKT) cells and mucosal-associated invariant T (MAIT) cells recognize lipids and vitamin B metabolites, respectively (3, 4). Once activated in a TCR-dependent and/or -independent manner, these unconventional T cells produce cytokines and some cytotoxic effector molecules, such as tumor necrosis factors (TNFs), perforin, and granzymes (Gzms) (5–8). Because these cells share properties of both the adaptive and innate responses, they are termed innate-like T cells, which express TCR with limited diversity and respond rapidly to relatively conserved antigen challenge, and can be said to represent a bridge between the adaptive and innate immune systems (8–11).

Like the intensively studied innate-like T cells, iNKT cells are restricted by CD1d and express a semi-invariant TCR Vα24-Jα18 in humans and Vα14-Jα18 in mice with a biased repertoire of Vβ chains (6, 12–15). In mice, iNKT cells are abundant in the liver, bone marrow and adipose tissue, but less in other tissues, such as spleen, blood and lung (16–18); however, in humans, they are rare in blood and liver compared to mice (19). Nevertheless, a recent study shows that iNKT cells are abundant in human omentum (20). iNKT cells recognize synthetic, self, and microbial lipid-based antigens presented by CD1d (6, 21, 22). Upon TCR or cytokine-mediated activation, iNKT cells rapidly produce cytokines and chemokines, exert cytotoxic activity by high expression of Gzms, perforin, and FasL, and regulate other immune cells (6, 23). As such, iNKT cells play important roles in combating infectious diseases and are also implicated in host responses to autoimmune diseases, chronic inflammatory diseases, and cancers (17, 24).

Mucosal-associated invariant T cells, a new subpopulation of innate-like T cells, have many similarities to iNKT cells, both in immunological properties and functions. Both cell types express semi-invariant TCRs, are restricted by monomorphic molecules, and can rapidly produce proinflammatory cytokines. With an increasing understanding of the antigens recognized by MAIT cells and their activation, the roles MAIT cells play in the immune response have been gradually revealed. Here, this review will summarize the discovery and phenotypic characterization of MAIT cells, then focus on MAIT cell antigen recognition and activation, and finally, discuss the roles of MAIT cells in infectious and non-infectious diseases.

## Mait Cell Discovery and Phenotype

In 1993, Porcelli et al. found that peripheral blood αβ CD4^−^CD8^−^ [double negative (DN)] T cells from healthy people preferentially expressed two invariant TCRα chains (15). One invariant TCRα chain, comprised of the Vα24 and Jα18 gene segments (Vα14 and Jα18 in mice), was expressed by what we now know as iNKT cells, while the other invariant TCRα chain used the Vα7.2 and Jα33 gene segments (Vα19 and Jα33 in mice) (12, 14, 15). Then, in 1999, a seminal study by Tilloy et al. showed that this invariant TCRα Vα7.2/Vα19-Jα33 chain was expressed by a new type of T cell subset present in humans, mice, and cattle, indicating conservation between mammalian species (25). In 2003, Treiner et al. observed that this novel subset of T cells was preferentially located in the intestinal lamina propria (LP) of humans and mice, and therefore, named these cells “mucosal-associated invariant T (MAIT) cells” (26). They also showed that MAIT cells were restricted by MR1, a monomorphic class I-related MHC molecule that is highly conserved in mammalian species (26, 27).

Although originally described as αβ DN T cells, MAIT cells, which are CD3^+^, may also be CD4^+^ and/or CD8^+^ (25, 28, 29). In humans, only 2–11% of MAIT cells in blood express CD4, while in Vα19iTg-Cα^−/−^ C57BL/6 (B6) mice, up to 50% of splenic MAIT cells are CD4^+^, implying different coreceptor requirements between humans and transgenic (Tg) mice (29). In wild-type (WT) mice (B6 or BALB/c), MAIT cells also consist of three subsets (DN, CD4^+^, and CD8^+^), and their frequencies vary in different tissues and strains (28). CD8^+^MAIT cells are present in different frequencies in humans and mice and are a mixture of CD8αα and CD8αβ cells (28–31). Contrary to iNKT cells, MAIT cells are abundant in humans, but rare in mice (17, 32). Moreover, as with iNKT cells, MAIT cells also display a restricted TCR repertoire. In humans, the majority of MAIT cells express a TCR comprised of a canonical Vα7.2-Jα33 TCRα rearrangement paired with limited TCRβ chains (predominantly Vβ2 or Vβ13), and less frequent usage of the non-canonical Vα7.2-Jα12/Jα20 TCRα rearrangement (15, 25, 29, 33). Mouse MAIT cell TCRs utilize an invariant Vα19-Jα33 TCRα chain predominantly paired with a Vβ6 or Vβ8 chain (25, 28). The variability of MAIT TCRβ chains may contribute to the functional heterogeneity of MAIT cells as immune effectors against various microbes (34).

Mucosal-associated invariant T cells express several cell surface proteins that can be used for identification and characterization (Table 1). Both human and mouse MAIT cells have a memory phenotype and may express transcription factors promyelocytic leukemia zinc finger (PLZF), retinoic acid-related orphan receptor γt (RORγt), and T-bet (28, 35–37). Detection of other cell surface proteins in various combinations has been used to detect MAIT cells. Both human and mouse MAIT cells express homing receptors, such as CXCR6, CCR9, α4β7, and/or CD103, which are consistent with their ability to migrate to the skin, liver, lung, and gut LP (28, 30). MAIT cells also express cytokine receptors, such as IL-7Rα, IL-12R, and IL-18Rα (28, 38–40).

**Table 1:** Surface phenotype of MAIT cells in mouse and human.

	MAIT cells			
	Vα19*i*Tg-Cα^−/−^ B6 mouse	WT B6 mouse	B6-MAIT^CAST^ mouse	Human
TCRα chain	Vα19-Jα33 (25, 41)	Vα19-Jα33 (25)	Vα19-Jα33 (25, 38)	Vα7.2-Jα33/20/12 (25, 29)

TCRβ chain	Predominantly Vβ8.1/8.2/6 (25, 41)	Predominantly Vβ8.1/8.2/6 (25, 28)	Predominantly Vβ8.1/8.2/6 (25, 38)	Predominantly Vβ2/13 (25, 29)

Coreceptor	CD8^lo^, DN, CD4 (29)	CD8, DN, CD4 (26, 28)	CD8^lo^, DN (38)	CD8αα, CD8αβ^lo^, DN, CD4^lo^ (29)

Egress from the thymus as	Naive (35, 41)	Naive (42)	n.d.	Naive (35, 42)

Phenotype in periphery	Naive (35, 41)	Effector memory (28, 36)	Memory (38)	Effector memory (35, 36)

Transcription factor	PLZF^lo^ (35)	PLZF, RORγt^hi^, T-bet^lo^ (28, 36, 37)	PLZF, RORγt (38)	PLZF, RORγt, T-bet (35, 36)

Activation marker	CD25, CD69, NK1.1 (11, 41, 43)	CD25, CD69^lo^, NK1.1^lo^ (28, 37, 38)	CD25, CD69 (38)	CD25, CD69, CD161^hi^ (11, 35, 39, 44)

Differentiation marker	CD44^lo^CD122^lo^ (35)	CD44^hi^CD62L^lo^ (28)	CD44^hi^ (38)	CD95^hi^CD62L^lo^ CD45RO^+^CD27^+^ (44)

Homing receptor	CXCR3, α4β1 (43)	CXCR6, CCR6, CCR9, CD103 (28)	CD103, CXCR6, CCR6 (38)	CCR2, CCR5, CCR6, CXCR6, CCR9, α4β7, CD103 (26, 30)

Cytokine receptor	n.d.	IL-7Rα, IL-18Rα, IL-12Rβ1 (28, 38)	IL-7Rα, IL-18Rα, IL-12Rβ1 (38)	IL-7Rα, IL-12R, IL-18Rα, IL-23R (30, 40, 45)

*n.d., not described*.

Initially, the reagent used to specifically identify and study human MAIT cells (CD3^+^CD161^hi^Vα7.2^+^T cells) was an antibody mix including anti-Vα7.2 and anti-CD161 mAb (35); however, because Vα7.2 TCR is also expressed by conventional T cells and CD1b-restricted germline-encoded, mycolyl lipid-reactive (GEM) T cells (Vα7.2-Jα9) (46, 47), an anti-Vα7.2 mAb alone is no longer considered an appropriate tool to study MAIT cells in humans. MAIT cells are phenotypically CD161^hi^ but CD161 is downregulated on MAIT cells in HIV patients (48), so CD161 is not a useful marker when examining specimens from HIV positive patients and potentially other cases. Because MAIT cells are rare in mice and there is no anti-Vα19 specific mAb available, researchers generated B6 Tg mice expressing only the TCR Vα19-Jα33 (Vα19i) chain to study MAIT cell development, phenotype, and antigen specificity (35, 41, 49). Unfortunately, maybe because of the developmental differences in MAIT cells between species, MAIT cells in Tg mice do not reflect the properties of MAIT cells in WT mice or humans (28, 41, 43). Recently, researchers have developed two new tools to study human and mouse MAIT cells: MR1-antigen tetramers (29, 50) and the B6-MAIT^CAST^ mouse, whose augmented MAIT cells are phenotypically very similar to human and WT mouse MAIT cells (Table 1) (38). These two new tools have facilitated the characterization of “natural” mouse MAIT cells, and the use of MR1-antigen tetramers circumvents the overlap/limitations of surrogate phenotypes to define MAIT cells in humans.

## Mait Cell Antigens

Mucosal-associated invariant T cells are known as non-conventional T cells in part because they recognize non-peptide antigens presented by the non-polymorphic MR1 molecule (51). MR1 has a standard MHC-I fold, and the antigen-presenting process of MR1 is similar to MHC-I and CD1d molecules, leading Huang et al. to speculate that MR1 could present peptide or lipid antigens to MAIT cells (52). Kjer-Nielsen et al. later showed that MAIT cells could recognize small molecule metabolites, including reduced 6-hydroxymethyl-8-d-ribityllumazine (rRL-6-CH_2_OH), 7-hydroxy-6-methyl-8-d-ribityllumazine (RL-6-Me-7-OH), and 6,7-dimethyl-8-d-ribityllumazine (RL-6,7-diMe), derived from the riboflavin [vitamin B2 (VB2)] biosynthetic pathway of bacteria (4). These compounds all have a bicyclic structure and a ribityl tail, but differ in their potency for activating MAIT cells (Table 2). Further research found that the most potent agonists were unstable pyrimidine intermediates, including 5-(2-oxoethylideneamino)-6-d-ribitylaminouracil (5-OE-RU) and 5-(2-oxopropylideneamino)-6-d-ribitylaminouracil (5-OP-RU), which are also derived from the VB2 biosynthetic pathway and are formed by the spontaneous non-enzymatic reactions between 5-amino-6-d-ribitylaminouracil (5-A-RU) and glyoxal or methylglyoxal, respectively (50). Although these three pyrimidines have the same single-ring structure and ribityl tail, 5-A-RU does not stimulate MAIT cells because MR1 is not refolded efficiently with 5-A-RU alone (50, 53). Because 5-OE-RU and 5-OP-RU are very unstable in water, Mak et al. preformed 5-OP-RU in DMSO-*d*_6_ and then diluted it in aqueous buffer, and as a result, improved its purity, quantity, and stability (54). They also designed and synthesized a new reagent **11**, a very potent analogue of 5-OP-RU, which was completely stable in water and showed a similar functional profile as 5-OP-RU, making it a valuable antigen analogue that can be used for MAIT cell research. Presently, six distinct antigens have been identified that are capable of activating MAIT cells, five VB2 metabolites and 5-OP-RU analogue. Although similar in structure, these antigens differ in their abilities to activate MAIT cells (Table 2) (4, 50, 54). In addition to these six agonists, MAIT cell inhibitors have also been identified, such as 6-formylpterin (6-FP) and acetyl-6-formylpterin (Ac-6-FP), which are folic acid [vitamin B9 (VB9)] derivatives with a bicyclic structure and a formyl group (4, 53, 55, 56); Ac-6-FP is a stronger inhibitor than 6-FP, which is the first MR1 ligand described (4, 53).

**Table 2:** The role of MR1 ligands in activation of MAIT cells.

Origin	Ligand	Role		Reference
VB2 intermediates	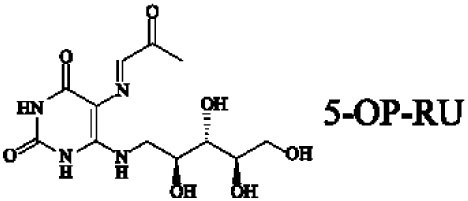	+++++	Agonist	(50, 53)
	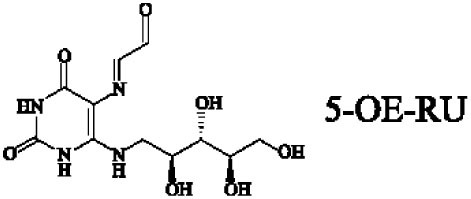	+++++		(50)
	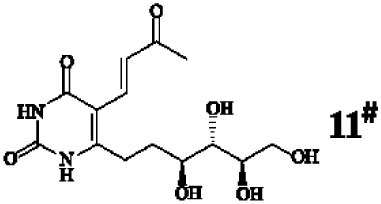	++++		(54)
	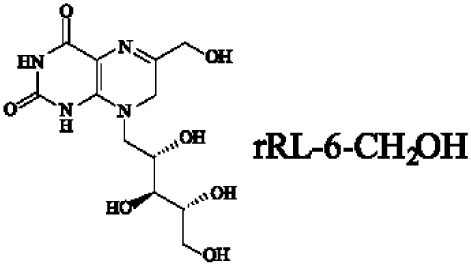	+++		(4, 53)
	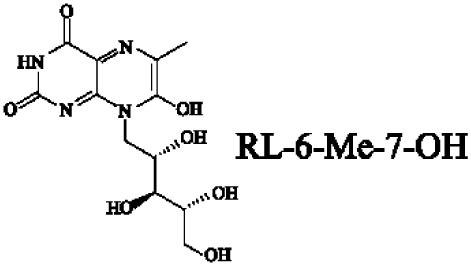	+		(4, 53)
	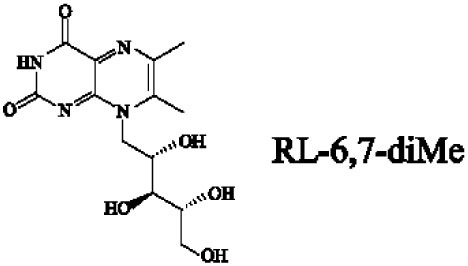	+		(4, 53)

VB9 derivatives	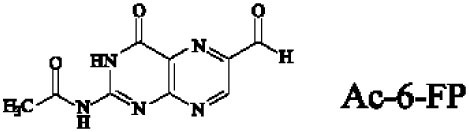	—	Inhibitor	(53)
	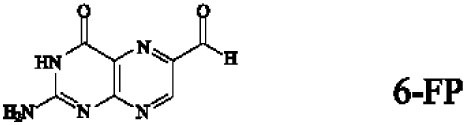	−		(4, 53)

Drugs and drug-like molecules	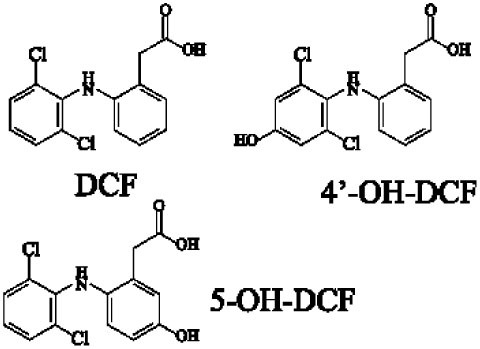		Agonist	(57)
	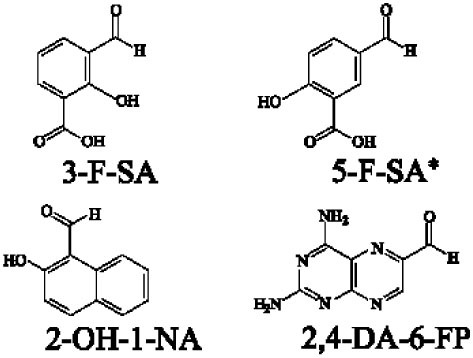		Inhibitor	(57)

*#: a potent analogue of 5-OP-RU*.

*: also as an agonist of activation of MAIT cells expressing a TRBV6-4.

*+: the potency for activating*.

*−: the potency for non-activating*.

Another reason MAIT cells are termed non-conventional is that they use a semi-invariant TCR and are restricted by a highly conserved MR1, implying a MAIT cell antigen repertoire with limited diversity (56). Known MAIT cell antigens only fill the A′ pocket of MR1 yet the heterogeneity of MAIT TCR chain Jα and Vβ usage suggested that MAIT cells may recognize additional antigens (17, 29, 30, 56). Therefore, finding more antigens will be helpful to understand MAIT cell recognition and activation. For example, the five known VB2 metabolite antigens, which are only synthesized by plants as well as many bacteria and yeast (56, 58), may reflect a primitive mechanism of self/non-self discrimination (59, 60). Recently, Keller et al. found that aside from VB9 derivatives and VB2 precursors, MR1 also presents drugs and drug-related molecules with diverse chemical structures (57), indicating that the pocket of MR1 has sufficient plasticity to accommodate a variety of molecules, including agonists or inhibitors of MAIT cell activation, such as diclofenac (DCF), 5-hydroxy DCF (5-OH-DCF) and 4′-hydroxy DCF (4′-OH-DCF), and 3-formylsalicylic acid (3-F-SA), 5-formylsalicylic acid (5-F-SA), 2-hydroxy-1-naphthaldehyde (2-OH-1-NA) and 2,4-diamino-6-formylpteridine (2,4-DA-6-FP), respectively. Therefore, we can modulate MAIT cell function by some of these drugs. Taken together, researchers have reported several agonists and inhibitors derived from VB metabolites and drugs or drug-related molecules. Of them, some molecules, such as DCF, DCF metabolites, and 5-F-SA, only activate specific subsets of MAIT cells with specific TCRβ chain usage (57), suggesting that the use of MAIT TCRβ chains “fine tunes” the responsiveness to certain antigens. Indeed, the effector responses of MAIT cells against *Escherichia coli* (*E. coli*) and *Candida albicans* (*C. albicans*) exhibit TCRβ chain bias. MAIT cells that are hyporesponsive to *E. coli* infection express Vβ8, Vβ13.1, and Vβ13.6, while MAIT cells that are responsive to *C. albicans* infection have a different TCRβ chain bias (34). To date, there are no reports about the nature of endogenous MAIT cell antigens; the antigens involved in MR1-mediated MAIT cell activation are all exogenous, which is inconsistent with iNKT cells (6, 24).

The identification of specific MAIT cell antigens has resulted in the generation of MR1-antigen tetramers. The first generation of tetramers was generated by loading of MR1 with rRL-6-CH_2_OH (29), which had lower affinity for staining MAIT cells. Now, however, the second generation of MR1 tetramers is prepared with 5-OP-RU, which is the most potent MAIT cell activator so far (50). Although MR1-antigen tetramers have facilitated studying and understanding mouse and human MAIT cell research, the use of MR1 tetramer staining has some limitations. Several authors have shown that MR1-antigen tetramer^+^ T cells are not all MAIT cells (10, 61) and contain 1–4% Vα7.2^−^ T cells, one subset of which detects infection with the riboflavin auxotroph *Streptococcus pyogenes* (*S. pyogenes*) in a TCR-dependent manner (61), implying that MR1 can present different ligands to MR1-antigen tetramer^+^ T cells depending on the TCRα chain usage. Indeed, Lepore et al. discovered a novel population of MR1-restricted T cells (MR1T), which are MR1-antigen tetramer^+^ T cells but are not MAIT cells, have variable TCRα chains, and can be activated by self-antigens (62). These antigens are purified from two tumor cell lines, and can activate MR1T cells without forming a Schiff base with MR1. However, the nature of self-antigens still need to be further studied.

## Mait TCR-MR1-Antigen Interactions

Several reviews are available that discuss MAIT TCR-MR1-antigen interactions (30, 51, 56, 59); therefore, we will focus on the difference between recently discovered antigens, drugs and drug-related metabolites, and riboflavin metabolites in interaction with TCR-MR1. Comparisons of the ternary structures of MAIT TCR-MR1-6-FP and MAIT TCR-MR1-RL-6-Me-7-OH suggested that MR1 has plasticity in its ability to accommodate ligands (59, 60). Consistent with this, there are more MR1 ligands discovered recently (50, 57). Whether it be VB2 metabolites or drugs and drug-related metabolites, the MAIT TCR docks with MR1-antigen complexes in an approximately orthogonal and central manner (50, 57, 63). However, based on crystallography studies, DCF and 5-OH-DCF presentation is dramatically different from that of VB2 antigens, which are presented in the MR1-binding cleft with the plane of the central phenylacetic acid ring basically perpendicular to that of the phenylacetic acid ring of DCF when it interacts with MR1-TCR (57).

Crystal structures of the MR1-antigen-MAIT TCR complex revealed that Tyr95α (Y95α) on MAIT TCRα chain interacts with ribityl tail of VB2 derived antigens, which plays a vital role in activating MAIT cells (29, 53, 63, 64); however, Y95α does not interact with DCF and 5-OH-DCF, which lack a ribityl group (57). DCF and 5-OH-DCF activate MAIT cells when the Y95α aromatic ring piles against the phenylacetic acid ring forming van der Waals forces with E99β from TCRβ chain (57). In contrast, inhibitors, such as 3-F-SA, 2-OH-NA, and 2,4-DA-6-FP, do not directly interact with the MAIT TCR (57), which is also the case with VB9-derived inhibitors (53). Therefore, small organic molecules acting as MAIT cell antigens directly contact the MAIT TCR, and vice versa.

## Mait Cell Activation

Mucosal-associated invariant T cells are known to be activated by some bacteria and yeasts, but not by viruses, in an MR1-dependent manner (11, 65). However, recent studies have shown that not only in viral diseases (7, 66), but also in some non-infectious diseases (67–69), MAIT cells can be activated in an MR1-independent manner. In either the MR1-dependent or -independent manner, upon activation, MAIT cells rapidly proliferate, secrete proinflammatory cytokines and other factors resulting in lysis of the infected cells, and have the capacity for B cell help (5, 70, 71).

**Figure 1:**
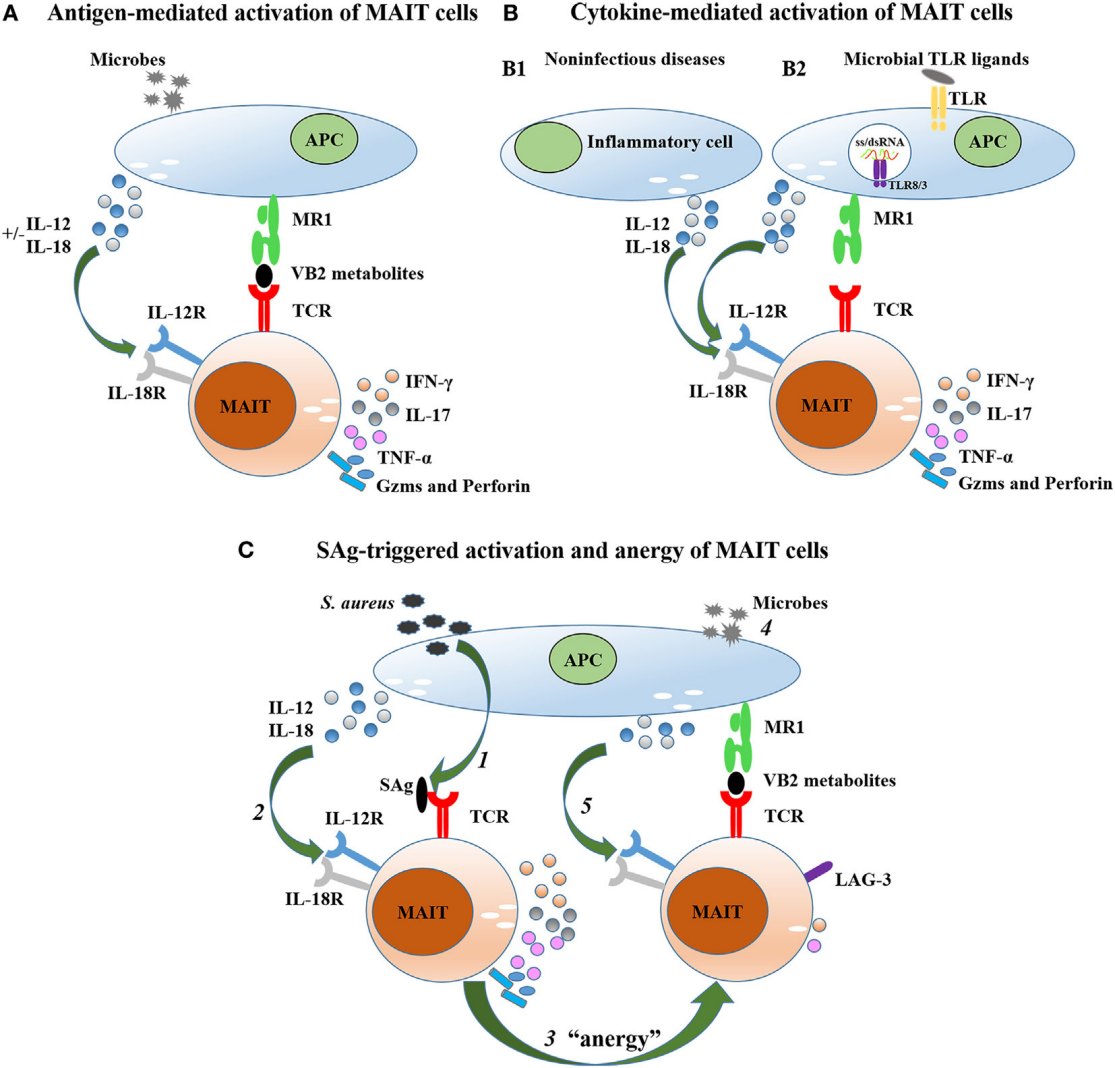
Mechanisms of mucosal-associated invariant T (MAIT) cell activation. **(A)** MAIT cells are activated by microbes that utilize a riboflavin biosynthetic pathway in an MR1-dependent manner. This activation can be enhanced when infected cells produce IL-12 and IL-18. **(B)** MAIT cells are activated by cytokines (IL-12 and IL-18) in an MR1-independent manner. These cytokines can be produced by inflammatory cells in non-infectious diseases (B1) or infected cells in viral disease (B2). **(C)** MAIT cells are activated by superantigen (SAg) in a T cell receptor (TCR)β-dependent manner (*1*) and/or cytokine-mediated (*2* and *5*) pathway, then upregulate inhibitory receptors such as lymphocyte-activation gene 3 (LAG-3), and then are anergized (*3*) to subsequent bacterial challenge (*4*).

Microbes which utilize the riboflavin biosynthetic pathway activate MAIT cells in an MR1-dependent manner (Figure 1A) (11, 37, 65, 72, 73). Human MAIT TCRs recognize VB2-derived antigens presented by MR1, and MAIT cells then upregulate the expression of CD25, CD69, and CD161, secrete Th1-type cytokines (IFN-γ and TNF-α) and Th17-type cytokines (IL-17 and IL-22), but do not secrete Th2-type cytokines (30, 40, 74), which is consistent with their expression of transcription factors like T-bet and RORγt (36, 44, 75). Mouse MAIT cells secrete high levels of IL-17, but lower levels of IL-4, IL-10, IL-13, IFN-γ, TNF-α, and GM-CSF upon activation with anti-CD3/CD28-coated beads (28, 30, 38). In addition to the secretion of proinflammatory cytokines, MAIT cells produce Gzms (GzmA, GzmK, and GzmB) and perforin, which function to effectively lyse infected cells (5, 76). Primary human MAIT cell activation is inefficient after *in vitro* stimulation with soluble ligands in an MR1-dependent manner, but also requires toll-like receptor (TLR) signaling and antigen-presenting cell (APC) activation (77). Consistent with this, accumulation and enrichment of MAIT cells *in vivo* not only requires VB2-derived antigens but also costimulatory signals, such as TLR agonists (37). Therefore, to establish a murine model of bacterial infection for MAIT cell studies, mice can be inoculated with synthetic antigens and TLR agonists such as CpG and poly I:C first, to promote MAIT cell accumulation and proliferation.

Like iNKT cells, MAIT cells can also be activated in an MR1-independent manner (Figure 1B). Several studies have shown that MR1-independent MAIT cell activation is dependent on IL-18 in synergy with other inflammatory mediators (7, 45, 66), which is consistent with the high expression of IL-18Rα on MAIT cells (30, 60). Upon cytokine-mediated activation in viral infections, MAIT cells can produce IFN-γ and GzmB, which can be suppressed or even abrogated by anti-IL-18 but not by MR1-blocking (7, 66, 78). In contrast, MAIT cells are activated via different mechanisms in response to different viral infections. In dengue virus (DENV) infection, MAIT cells respond to both IL-12 and IL-18, while in hepatitis C virus (HCV) and influenza virus (IAV) infection, MAIT cell activation is predominantly dependent on IL-18, which is produced by CD14^+^ monocytes (66, 75, 78). Moreover, IFN-α/β may also play a role in MAIT cell activation during viral infection (7). Corresponding to activation of MAIT cells in viral infection, previous studies showed that TLR8 and TLR3 are potent activators of MAIT cells, promoting the secretion of IL-12 and IL-18 by APCs (7, 45, 79). In addition to viral infections, MAIT cells also respond to infections with some bacterial species, such as *Mycobacterium tuberculosis, Mycobacterium bovis* bacillus Calmette–Guérin, and *Enterococcus faecalis*, in an MR1-independent manner (45, 80, 81). So, although there are two pathways of MAIT cell activation in response to bacterial infections, MR1-dependent and MR1-independent, the relative contribution of the two pathways is still unclear. Interestingly, MAIT cells are also activated in some non-infectious diseases. In patients with systemic lupus erythematosus (SLE), MAIT cells can be activated by IFN-α, IL-15, and IL-12 plus IL-18 in the absence of exogenous antigens (69). The positive correlation between the plasma concentration of these cytokines and the expression levels of CD69 on MAIT cells suggests that proinflammatory cytokines may activate MAIT cells and may play a role in the pathogenesis of SLE and possibly other inflammatory processes.

Interestingly, recently a new study showed that, apart from MR1-dependent and cytokine-mediated activation, MAIT cells can also be activated by superantigens (SAgs) produced by *Staphylococcus aureus* and *S. pyogenes* in a TCR Vβ-dependent manner (Figure 1C) (82), following which Sandberg et al. wrote a commentary to highlight this new discovery (83). Moreover, SAgs also activate MAIT cells through IL-18/IL-12 signaling, which is dominant over the TCR Vβ-dependent pathway of MAIT cell activation (Figure 1C). MAIT cell activation also requires MHC-II interaction with SAgs, which can activate conventional T cells through binding to TCR Vβ chains, and conventional T cells then promote the production of IL-18 and IL-12 through release of inflammatory mediators (82, 84). Upon activation by SAgs, MAIT cells make significant contributions to the cytokine storm via rapid production of proinflammatory cytokines but then are anergized to subsequent bacterial challenge through upregulation of inhibitory receptors such as lymphocyte-activation gene 3, demonstrating that MAIT cells also play a role in pathogenesis in some bacterial infection (82, 83).

## Mait Cells and Diseases

In 2010, two studies reported that MAIT cells reacted to infected cells (11, 65). Since that time, there has been a growing body of research describing the role of MAIT cells in disease. Many have suggested that MAIT cells play important roles in infectious diseases, including bacterial and viral diseases, and non-infectious diseases, including autoimmune diseases and cancer; this topic has been reviewed recently (42, 74, 85–88), so here, we will focus on more recently published articles.

Many studies have described a role of MAIT cells in bacterial infections (38, 89). For example, human CD8^+^MAIT cells are important in combating *Salmonella enterica serovar Typhi* (*S. typhi*) infection (73) and recently using a mouse model of *Salmonella* Typhimurium infection, MAIT cells have been shown to accumulate in the lungs of infected mice (37). Similarly, in response to *S. typhi*, MAIT cells are activated and home to affected tissues, such as the gut (73). MAIT cells can also be activated and functionally impaired in patients with scrub typhus, caused by *Orientia tsutsugamushi* (90).

As mentioned above, MAIT cells may also be involved in the clearance of some viral infections (45, 80). In patients with HCV and DENV infections, MAIT cells are present at a lower frequency in blood than in healthy controls, and can be activated in a cytokine-mediated manner to upregulate the expression of IFN-γ and GzmB (7, 78, 91). Furthermore, there is a negative correlation between the degree of depletion of the intrahepatic MAIT cells and the severity of liver inflammation and fibrosis in HCV-infected patients (78). In IAV infections, human MAIT cells can resist infection by IL-18-dependent activation (7, 66). Moreover, the function and frequency of MAIT cells can be impaired and reduced, respectively, in human T lymphotropic virus type 1 infection (92).

Apart from infectious diseases, MAIT cells also play potential roles in inflammatory diseases and cancers. Patients with ankylosing spondylitis have a lower frequency of MAIT cells in peripheral blood but a higher frequency in synovial fluid (SF) compared with the healthy controls, and MAIT cells in SF of patients with an exaggerated IL-17 phenotype are primed by IL-7 (93). Moreover, human MAIT cells are reduced and functionally altered in the peripheral blood of patients with common variable immunodeficiency (CVID) (94) and primary Sjogren’s syndrome (pSS) (68); however, the alteration of MAIT cell function in CVID and pSS patients is different. In CVID patients, MAIT cells are more activated and produce cytokines at higher frequencies than MAIT cells in healthy controls, while in pSS patients, MAIT cells are activated at a lower level and produce less cytokines than MAIT cells in healthy controls. In some cancers, such as gastric, colon, and lung cancers, MAIT cells can migrate from the peripheral blood to affected tissues, express GzmB and perforin, and thus have the potential to kill cancer cells in patients with mucosal-associated cancers (67). Moreover, MAIT cells heavily infiltrate the hepatic metastases in patients with colorectal carcinoma (CRC), and the function of tumor-infiltrating and tumor-margin MAIT cells are impaired irrespective of preoperative chemotherapy, implying the attractiveness of therapeutic targeting of MAIT cells in CRC (95).

However, MAIT cells also play a pathogenic, not protective, role in SAg-mediated illnesses (82, 83). MAIT cells activated by SAgs not only contribute to cytokine storm but also acquire an anergic phenotype, indicating a role for them in immunopathology and immunosuppression and also implying that MAIT cells can be used as an efficacious therapeutic target of SAg-mediated illnesses. Collectively, based on an ever increasing understanding of the mechanisms involved in MAIT cell activation, MAIT cells continue to be implicated in more and more diseases, often with differing mechanisms of activation.

Given their ability to rapidly produce cytokines after stimulation, direct lysing of microbe-infected cells, and their potential to regulate immune responses, MAIT cells similar to iNKT cells and Vγ9Vδ2 T cells, which have been used as targets in immunotherapy research (96–101), may also be an effective target for disease treatment. For example, due to their potential role in cancers and infectious diseases, adoptive MAIT cell transfer therapy may be a promising approach to treat diseases (102). Moreover, with a growing number of MAIT cell antigens to be found, the potent antigens can be administered alone or pulsed on APCs to enhance immune responses, or can be used as an effective adjuvant to boost vaccine efficacy. Furthermore, as their pathogenic role in SAg-mediated illnesses, blocking the function of MAIT cells may alleviate the pathological damage caused by SAgs (82). However, there is still no one immunotherapy based on MAIT cells to be used in clinical trials. Therefore, our better understanding of the biology of MAIT cells will accelerate the use of MAIT cell immunotherapy in diseases.

## Conclusion

Since the original description in 1993 of a population of unconventional DN T cells with a limited repertoire, much has been learned about the role of MAIT cells in immune response, including a better understanding of MAIT cell phenotypes, restricting molecules, MAIT cell development, antigen recognition, molecular interactions of the TCR-MR1-antigen complex, and their roles in diseases. It was previously believed that in human T cells expressing Vα7.2-Jα33^+^ TCRα chain were exclusively MAIT cells, but later it was found that, in addition to canonical Vα7.2-Jα33 TCRα, MAIT cells also express non-canonical Vα7.2-Jα12/20 combinations (29). Similarly, it was originally believed that MR1-restricted T cells were MAIT cells, but later it was confirmed that MR1-restricted T cells are not all MAIT cells, which also contain Vα7.2^−^ T cells (10, 61). Presently MAIT cells are defined as those T cells which express a Vα7.2 TCRα chain and are restricted by MR1. It is now well known that MAIT cell antigens are not only confined to VB2 metabolites. Drugs and drug-related molecules can also serve as MAIT cell agonists (57). MAIT cell activation can also be triggered by SAgs in an MR1-independent manner, but dependent on the certain TCR Vβ and/or cytokine-mediated pathway (82, 83).

With more and more antigens being described that are recognized by MAIT cells, along with new and different mechanisms of activation, our understanding of MAIT cells continues to expand. However, some questions still remain unanswered. Firstly, although the number of exogenous antigens continues to grow, are there endogenous antigens that activate MAIT cells? And what is their nature? Secondly, even though the intracellular trafficking pathway of the MR1-antigen complexes within the cell has been shown (103, 104), what intracellular machinery is responsible for retaining empty MR1 molecule in the endoplasmic reticulum to load the extracellular antigens? Thirdly, as MAIT cells are recognized as a potential subset of tissue-resident T cells, what is their contribution to the pathogenesis of tissue or organ (such as liver and lung) diseases? Although some work has described MAIT cells in animals other than human and mouse, what are the roles of MAIT cells play in other animal species? Fourthly, how can MAIT cell responses be modulated, either through small molecules or immunotherapy, to improve human health? Finally, almost all published human data are generated by *in vitro* assays with a short period of experimental time. Given all the caveats of any *in vitro* experiments, the findings/observations remain to be verified by *in vivo* studies in mouse work, albeit the species difference.

Mucosal-associated invariant T cells, the once obscure cell population, represent up to 10% of T cells in the human peripheral blood, therefore, it is imperative that we continue to identify and characterize antigens and mechanisms that activate MAIT cells to better understand the whole scope of the immune response against microorganisms and disease pathogenesis. New insights will lead to improved therapeutics for infectious and non-infectious diseases controlled in part by MAIT cells.

## Author Contributions

JC designed the outline, organized the text, and critically revised the manuscript. XX drafted the manuscript. All authors reviewed and approved the final version of the manuscript.

## Conflict of Interest Statement

The authors declare that the research was conducted in the absence of any commercial or financial relationships that could be construed as a potential conflict of interest.
